# Novel anti-HER2 monoclonal antibodies: synergy and antagonism with tumor necrosis factor-α

**DOI:** 10.1186/1471-2407-12-450

**Published:** 2012-10-04

**Authors:** Ceyhan Ceran, Murat Cokol, Sultan Cingoz, Ipek Tasan, Mehmet Ozturk, Tamer Yagci

**Affiliations:** 1 BilGen Genetics and Biotechnology Research Center, Department of Molecular Biology and Genetics, Bilkent University, Ankara, Turkey; 2 Biological Sciences and Bioengineering Program, Faculty of Engineering and Natural Sciences, Sabanci University, 34956, Istanbul, Turkey; 3 Department of Medical Biology, Dokuz Eylul University Medical School, Izmir, Turkey; 4 Faculty of Science, Department of Molecular Biology and Genetics, Gebze Institute of Technology, 41400, Kocaeli, Turkey

**Keywords:** HER2, ERBB2, TNF-α, Monoclonal Antibodies, Epitope Mapping, Growth Inhibition, Breast Cancer, Synergy, Antagonism

## Abstract

**Background:**

One-third of breast cancers display amplifications of the *ERBB2* gene encoding the HER2 kinase receptor. Trastuzumab, a humanized antibody directed against an epitope on subdomain IV of the extracellular domain of HER2 is used for therapy of HER2-overexpressing mammary tumors. However, many tumors are either natively resistant or acquire resistance against Trastuzumab. Antibodies directed to different epitopes on the extracellular domain of HER2 are promising candidates for replacement or combinatorial therapy. For example, Pertuzumab that binds to subdomain II of HER2 extracellular domain and inhibits receptor dimerization is under clinical trial. Alternative antibodies directed to novel HER2 epitopes may serve as additional tools for breast cancer therapy. Our aim was to generate novel anti-HER2 monoclonal antibodies inhibiting the growth of breast cancer cells, either alone or in combination with tumor necrosis factor-α (TNF-α).

**Methods:**

Mice were immunized against SK-BR-3 cells and recombinant HER2 extracellular domain protein to produce monoclonal antibodies. Anti-HER2 antibodies were characterized with breast cancer cell lines using immunofluorescence, flow cytometry, immunoprecipitation, western blot techniques. Antibody epitopes were localized using plasmids encoding recombinant HER2 protein variants. Antibodies, either alone or in combination with TNF-α, were tested for their effects on breast cancer cell proliferation.

**Results:**

We produced five new anti-HER2 monoclonal antibodies, all directed against conformational epitope or epitopes restricted to the native form of the extracellular domain. When tested alone, some antibodies inhibited modestly but significantly the growth of SK-BR-3, BT-474 and MDA-MB-361 cells displaying *ERBB2* amplification. They had no detectable effect on MCF-7 and T47D cells lacking *ERBB2* amplification. When tested in combination with TNF-α, antibodies acted synergistically on SK-BR-3 cells, but antagonistically on BT-474 cells. A representative anti-HER2 antibody inhibited Akt and ERK1/2 phosphorylation leading to cyclin D1 accumulation and growth arrest in SK-BR-3 cells, independently from TNF-α.

**Conclusions:**

Novel antibodies against extracellular domain of HER2 may serve as potent anti-cancer bioactive molecules. Cell-dependent synergy and antagonism between anti-HER2 antibodies and TNF-α provide evidence for a complex interplay between HER2 and TNF-α signaling pathways. Such complexity may drastically affect the outcome of HER2-directed therapeutic interventions.

## Background

With a lifetime risk of ~12%, breast cancer is the most common cancer in women [1]. Although the survival rates of breast cancer patients are increasing due to the recently developed therapeutic approaches, breast cancer remains the most frequent cause of cancer-related death in women [2]. More than 225,000 new cases of female-breast cancer are estimated to occur in 2012, in the United States alone, and 40,000 female deaths are expected as a result [3]. 25-30% of breast cancers display HER2 overexpression [4]. *ERBB2* gene amplification that was discovered in the early 1980s is the main cause of HER2 overexpression [5]. HER2 is a tyrosine kinase receptor belonging to the ErbB/HER family that also includes epidermal growth factor receptor (EGFR/HER1), ErbB3/HER3 and ErbB4/HER4. HER2 displays tyrosine kinase activity, but has no known ligand. ErbB family receptors are activated by homodimerization or heterodimerization. HER2 is activated either by ligand-independent homodimerization when it is overexpressed, or by heterodimerization with a ligand-dependent ErbB/HER member, in particular with ErbB3/HER3 [6].

Following the discovery of *ERBB2* gene amplifications in breast cancer, the HER2 receptor became an attractive target for monoclonal antibody-based therapies. Trastuzumab is the first clinically approved humanized monoclonal antibody targeting HER2. It produced 11-26% overall response rates as monotherapy for metastatic breast cancer. In addition, as adjuvant to chemotherapy, Trastuzumab significantly increased disease-free and overall survival in both early stage and metastatic breast cancers. Trastuzumab is effective only in HER2-amplified/overexpressing tumors and primary resistance to therapy is a challenge [7]. Moreover, the majority of metastatic breast cancer patients who initially respond to Trastuzumab begin to demonstrate disease progression again within 1 year [8]. Trastuzumab resistance was linked to many factors, including the overexpression of other ErbB/HER family receptors and/or their ligands [9,10], insulin-like growth factor receptors [11,12], MUC4 [13] and soluble extracellular domain (ECD) of HER2 circulating in the blood [14,15], activating PI3K mutations [16], PTEN deletions [17], downregulation of cyclin-dependent kinase inhibitor p27 [18] and increased Akt activity [19,20].

HER2-HER3 heterodimer is known to be the most potent activated form of HER2 in terms of strength of interaction, ligand-induced tyrosine phosphorylation and down-stream signaling. Indeed, HER3 might be a necessary partner for HER2-mediated oncogenic activity in HER2-overexpressing tumors [6]. The ECD of HER2 is composed of four subdomains and Trastuzumab is directed to its subdomain IV [21]. The antibody inhibits HER2 activation [6], but cannot prevent HER2-HER3 heterodimerization [22]. Pertuzumab is another antibody directed to another epitope that is located at subdomain II, which is involved in receptor dimerization [23]. It appears to be more effective than Trastuzumab, in particular in low HER2-expressing tumor cells because of its ability to interfere with HER2-HER3 heterodimerization [23]. Pertuzumab and Trastuzumab were shown to act synergistically against breast cancer cell survival [24], suggesting that antibodies against different epitopes of the ECD are able to generate not only additive but also synergistic cellular responses. The limited efficacy of Trastuzumab for cancer therapy, problems of acquired resistance and the potential impact of novel anti-HER2 antibodies as therapeutic partners or alternative therapeutics justify more investment on HER2 targeting with novel monoclonal antibodies. Here, we describe five novel monoclonal antibodies directed against an epitope (or epitopes) restricted to the native conformation of HER2 ECD. *In vitro* studies provide evidence for anti-cancer activity of these antibodies. More interestingly, these novel antibodies appear to interact with tumor necrosis factor-α (TNF-α) in a cell-dependent manner, generating both synergistic and antagonistic growth responses.

## Methods

### Reagents

A disulfide-linked homodimer of histidine-tagged extracellular domain of HER2 fused to human IgG1 Fc domain (HER2 ECD), a similar construct based on EGFR extracellular domain (EGFR ECD), and IgG1 isotype control antibody were from R&D Systems (MN, USA). Alkaline phosphatase (AP)- and horseradish peroxidase (HRP)-conjugated secondary antibodies to mouse and human IgGs, propidium iodide, trichloroacetic acid (TCA), Sulforhodamine B (SRB), Incomplete Freund’s Adjuvant, anti-calnexin antibody (#C4731), *p*-nitrophenyl phosphate, disodium salt (PNPP), 4', 6-diamidino-2-phenylindole (DAPI), insulin and Bromodeoxyuridine (BrdU) were from Sigma Aldrich (MO, USA). Anti-HER2 CB11antibody (#ab8054) was from Abcam (MA, USA). Anti-BrdU antibody (#M0744) was from Dako (Glostrup, Denmark). Anti-Akt (#9272), anti-phospho-Akt (#9271), anti-Erk 1/2 (#9107), anti-phospho-Erk 1/2 (#9106) antibodies were from Cell Signaling (MA, USA). Anti-phospho-HER2 (#sc-1011694) and anti-Cyclin D1 (#sc-246) antibodies were from Santa Cruz (California, USA). Alexa Fluor 488-conjugated secondary antibodies to mouse and human IgGs and Lipofectamine 2000 were from Invitrogen (CA, USA). HiTrap Protein G HP columns and ECL plus western blot detection kit were from GE-Healthcare (WI, USA). Recombinant human TNF-α was from PeproTech (NJ, USA). RNAse A and all restriction enzymes were from Fermentas (Vilnius, Lithuania). Trastuzumab (Herceptin), EDTA-free protease inhibitor cocktail tablets and phosSTOP (phosphatase inhibitor cocktail) tablets were from Roche Ltd (Basel, Switzerland). Antibody isotyping kit was from Thermo Fisher Scientific Inc (IL, USA). Protein G coupled Sepharose gel slurry was from East Coast Biologics (ME, USA).

### Cell culture

SK-BR-3, MDA-MB-361, T47D, MCF-7 and BT-474 cell lines were obtained from ATCC. Huh-7 cell line is used in lab since 1995 and was last tested for authenticity in 2010 (originally from Jack Wands Lab at Massachusetts General Hospital, Boston, MA). All breast cancer cell lines have been tested and authenticated by short tandem repeat profiling in September 2009 and February 2012, as described previously [25]. SK-BR-3 cells were grown in McCoy’s 5A medium. MDA-MB-361, T47D, MCF-7, BT-474 and Huh-7 cells were grown in Dulbecco’s Modified Eagle Medium (DMEM), as described [25,26]. Hybridomas were grown in RPMI 1640, DMEM or Serum Free Media (SFM) from Hyclone (IL, USA). Cell culture media were purchased from GIBCO (CA, USA), Sigma or Hyclone, and were supplemented with 10% fetal calf serum (FCS), 1% penicillin/streptomycin (P/S), 1% non-essential amino acids and 1% L-glutamine, unless otherwise stated. BT-474 cell growth medium was supplemented additionally with 10 μg/ml insulin.

### Fluorescent in situ hybridization analysis of *ERBB2* copy numbers

MCF-7, T47D, MDA-MB-361, SK-BR-3 and BT-474 cells were harvested at confluence and fixed in Carnoy’s fixative (3:1 methanol/glacial acetic acid). A dual-color FISH was performed with Texas Red-labeled *ERBB2* and FITC-labeled chromosome 17 α-satellite (D17Z1) DNA probes mix (LPS 001; Cytocell Ltd, Cambridge, UK), as described by supplier with minor modifications. The slides were treated at 72°C for 2 min in denaturation solution containing 70% formamide, 2XSSC (3 M NaCl, 0.3 M sodium citrate, pH 7.0). The probe mixture was applied onto slides; the area was covered with a 22X22 mm glass coverslip, sealed with rubber cement. Slides were placed on a hot plate and heated at 75°C for 5 min. The hybridization was done overnight at 37°C in humidified chambers. Post-hybridization washes were done once with 0.4XSSC at 72°C for 2 min and once with 2XSSC containing 0.05% Tween 20, at room temperature for 30 s. After post-hybridization washes, the slides were dehydrated in a series of 70%, 85%, and 97% ethanol. Air-dried slides were counterstained with 4,6-diamidino-2-phenylindole (DAPI) in an anti-fade solution. Copy numbers in >100 nuclei of each cell line were counted under a Nikon Eclipse E600 epifluorescence microscope (Nikon Corp., Tokyo, Japan) equipped with filter sets appropriate for DAPI, FITC, and rhodamine. Individual single-color images of DAPI, FITC, and rhodamine were acquired through a high-sensitivity monochrome charge-coupled device (CCD) camera integrated to a Power Macintosh. Digitally acquired individual images were overlaid and operated with MacProbe image analysis software (PCI Scientific Systems League City, TX, USA). *ERBB2* copy numbers and ploidy level based on average counts of centromere 17 in interphase and metaphase FISH were evaluated according to international standard cytogenetic nomenclature [27].

### Generation of anti-HER2 monoclonal antibodies

Animal experiments described here have been pre-approved by the Bilkent University Animal Experiments Ethical Review Panel, and conducted at Bilkent Animal House, which is certified by the Ministry of Agriculture of Turkey. All procedures complied with the guidelines of the Ministry of Agriculture (Official Gazette. No. 25464). BALB/c mice were immunized five times with live SK-BR-3 cells (5–10 × 10^6^ cells in PBS) by intra-peritoneal injection at three-week intervals. As a final booster, each mouse received 20 μg recombinant HER2 ECD protein injected with incomplete Freund’s adjuvant. On the third day of last immunization, mice were sacrificed and their splenocytes were fused with SP2/O myeloma cells to produce hybridomas as previously described [28].

### HER2 ECD ELISA assay

96-well Polysorb (NUNC, Roskilde, Denmark) microtiter plates were coated overnight at 4°C with HER2-ECD (25–50 ng/well) in carbonate buffer. Following saturation with 1% BSA for one hour, plates were incubated with hybridoma supernatants or purified primary antibodies for 2–4 h, and bound antibodies were detected by further incubation with AP-conjugated anti-mouse IgG antibodies. Trastuzumab binding was tested using AP-conjugated anti-human IgG antibodies. Phosphatase activity was measured using PNPP substrate with absorbance (OD) reading at 405 nm using μQuant ELISA reader (BioTek, VT USA). The cross-reactivity tests with EGFR ECD were performed under the same conditions, except that ELISA plates were coated with EGFR ECD.

### Antibody isotyping and affinity purification

The isotypes of monoclonal antibodies were determined using a commercial kit. Selected hybridoma clones were expanded and the antibodies were purified from the culture supernatants by FPLC using HiTrap Protein G HP columns (GE Healthcare, WI, USA).

### Immunofluorescence

Cells were seeded on coverslips in 6-well plates. After 36 h of cell culture, they were fixed with 2% paraformaldehyde and permeabilized with 0.1% Triton X-100. Following saturation with 10% FCS, the fixed cells were incubated with anti-HER2 antibodies for 90 min. Primary antibody binding was detected using Alexa Fluor 488 conjugated goat anti-mouse IgG or Alexa Fluor 488 conjugated goat anti-human IgG antibodies, depending on the type of the primary antibody. Nuclei were counterstained with DAPI and cells were observed using Zeiss Axio Imager.A1 microscope (Jena, Germany).

### Flow Cytometry

Cells (3-5×10^5^) were fixed with 2% paraformaldehyde and incubated 1 h with 5 μg/ml anti-HER2 antibodies or IgG1 isotype control antibody (in 1% BSA and 0.5 mg/ml NaN_3_ in PBS). Cells were then incubated 1 h with Alexa-488-conjugated anti-mouse IgG antibody and the percentage of positively stained cells were counted on FACSCalibur (BD Biosciences).

### Immunoprecipitation

For detection of endogenous HER2 protein, cells were lysed in RIPA buffer (150 mM NaCl, 50 mM Tris–HCl pH; 7.4, 1 mM EDTA, 1% Triton X-100, 1% Na-deoxycholate, 0.1% SDS, 1X Protease Inhibitors) [29,30], 2 mg protein were diluted with milder NP40 buffer (150 mM NaCl, 50 mM Tris–HCl pH: 8, 1% NP40, 1X Protease Inhibitors) and incubated with 20 μg antibody for 2 h on a rotator at 4°C. Then, 200 μl Protein G-coupled Sepharose gel slurry was added, and the mixture was incubated 4 h at 4°C.

### Western blot

Following immunoprecipitation experiments, antigen-antibody bound Protein G beads were boiled in SDS-loading buffer; solubilized proteins were run on 8% or 4-12% gradient SDS-PAGE. Proteins subjected to SDS-PAGE were transferred to PVDF membrane using XCell II Blot Module (Invitrogen, CA, USA). Unoccupied protein binding sites on the PVDF membrane were saturated by incubating with 5% dried milk (in TBS-T) for 1 h. Full length and truncated forms of HER2 protein were detected using CB11 antibody directed against intracellular domain. Briefly, membranes were incubated overnight with CB11 antibody (1/500 dilution), followed by 1 h incubation with HRP-conjugated secondary antibodies to mouse IgGs and bound antibody was detected using ECL plus western blot detection kit.

### Epitope mapping

The experiment was based on testing of antibody binding to HER2 proteins that have been modified by deletion of different subdomains of its ECD. Mammalian expression plasmids were constructed and transiently transfected into Huh-7 cells. Antibody binding was tested by combined immunoprecipitation-western blot and indirect immunofluorescence assays. A pDEST26-derived plasmid vector expressing full-length human HER2 protein (OCABo5050G0219D; shortly called p219D here) was obtained from Source BioScience LifeSciences (Nottingham, UK). HER2 extracellular domain was extracted from p219D by Sac I/Eco RI digestion leaving the coding sequence for the N-terminal 22 amino acid signal peptide intact. pCC2001 was produced by inserting Not I and Xho I sites between this sequence and the sequence encoding the rest of HER2 protein starting from the transmembrane domain. DNA sequences allowing the inclusion of domains I, II, III and IV of ECD under different combinations were amplified by PCR and inserted into pCC2001 plasmid using the Not I and Xho I sites. The integrity of cloned sequences has been verified by DNA sequencing. Huh-7 cells (6×10^5^) were transfected in 6-well plates with different HER2 plasmid constructs (5 μg each) using Lipofectamine 2000, following a protocol provided by the manufacturer. Following 8-h Lipofectamine incubation, cells were washed with PBS and incubated overnight with fresh complete media (without P/S). Next day, the cells were trypsinized and replated. On the second day of cell-expansion, the cells were collected, lysed in RIPA buffer and subjected to immunoprecipitation and western blot analyses as described above, except that 150 μg proteins were immunoprecipitated using 15 μg antibody.

### Cell growth assay

This assay was performed as described previously [31]. Briefly, cells (10^3^) were seeded in 96-well plates in triplicate and allowed to attach by 2 h of preincubation. Antibodies were diluted in cell culture medium and added to preincubated cells to obtain 0–20 μg/ml concentrations under a final volume of 200 μl medium/well. Cells were then incubated up to 6 days. TNF-α treatment was performed similarly except that cells were preincubated for 6 h. For antibody and TNF-α co-treatment experiments and checkerboard assays, cells were preincubated for 2 h for attachment, followed by 4-h incubation with antibodies prior to TNF-α addition. At the end of each time point, culture supernatants were discarded. Attached cells were washed twice with PBS, fixed with ice-cold 10% TCA for one hour, followed by Sulforhodamine B-based reading, as described [32].

### Drug interaction modeling

Drug interaction scores for pairwise combinations of TNF-α with individual antibodies at fixed concentrations were calculated as follows. Growth level in a drug condition was defined as the growth measurement in the drug condition normalized by growth measurement under no drug. To understand the interaction between an antibody and TNF-α at certain concentrations, growth levels under single antibody, TNF-α, antibody + TNF-α were compared. Expected growth level under no drug interaction was defined for each pairwise drug combination as the multiplication of the growth levels under each individual drug, following the Bliss Independence Model for drug interactions [33]. The observed growth level for each combination was divided by the expected growth level to find an interaction score. According to this analysis, a score of 1 means no interaction, or independence, a score smaller than 1 means synergy, and a score larger than 1 means antagonism. For the drug interaction experiments where more than one concentration combination was experimentally tested, a single interaction score between two drugs was computed. For this, a modified version of the above algorithm and the data from all concentration combinations was used, as described previously [34]. The scores and the scale of these scores are equivalent to the single combination drug interaction experiment and are interpreted in the same way (<1 = synergy; >1 = antagonism). Each interaction experiment was done in triplicate, and the average growth levels were used for interaction score calculation.

### Bromodeoxyuridine incorporation assay

Cells (10^4^) were plated on coverslips in 12-well plates, and treated as described for cell growth assays. At 48^th^ hour, BrdU (10 μg/ml) was added into cell culture media and cells were further incubated for 24 h. The detection of BrdU incorporation was performed as described previously [35], except that Alexa Fluor 488-conjugated anti-mouse IgG antibody was used for visualization. Cell nuclei were counterstained with DAPI, examined using Zeiss Axio Imager.A1 microscope and representative photographs (3–5 frames per coverslip) were acquired. Percent BrdU incorporation was calculated after manual counting of BrdU-positive (BrdU^+^) and BrdU-negative (BrdU^-^) nuclei.

### Cell cycle analysis

Analyses were performed as described previously [36]. Briefly, 2.5×10^4^ (for 6-day samples) or 10^5^ (for 1 and 3-day samples) SK-BR-3 cells were seeded in 6-well dishes in McCoy’s 5A medium. After 36 h incubation, the medium was replaced with fresh McCoy’s 5A medium including BH1 or IgG1 isotype control antibody (final 5 μg/ml) ± 1000Unit/ml TNF-α. On days 1, 3 and 6 after the addition of antibodies, both suspended and adherent cells were collected, fixed and stained with propidium iodide (PI). PI-stained cells were measured and analysed by flow cytometry.

### Analysis of the downstream protein expression

In order to analyze the effect of BH1 treatment on the downstream pathway, SK-BR-3 cells were treated for 1–24 h with 10 μg/ml BH1, 1000 Unit/ml TNF-α alone or their combination. Cells collected at each time point (1^st^, 2^nd^, 4^th^, 8^th^, 12^th^, 24^th^ hour) were lysed with RIPA buffer (containing 1X PhosSTOP). Solubilized proteins were resolved by SDS-PAGE and analyzed by immunoblotting. PVDF membranes initially used for detection of phospho-Akt, phospho-Erk 1/2 and phospho-HER2 proteins were stripped once (62.5 mM Tris-Cl pH: 6.7, 2% SDS, 100 mM β-mercaptoethanol), washed thoroughly with TBS-T and re-blotted with anti-Akt, anti-Erk 1/2, anti-cyclin D1 and anti-calnexin antibodies. Following incubation with HRP-conjugated secondary antibodies to mouse or rabbit IgGs, bound antibodies were detected using ECL plus western blot detection kit.

### Statistical analysis

Experimental data were presented as mean ± SD. Statistical analysis of experiments that have been performed at least in triplicate was done using Student’s *t* test.

## Results

### Novel monoclonal antibodies directed to extracellular domain of HER2

We used a combined immunization protocol with repeated injections of live SK-BR-3 cells to BALB/c mice, followed by a single injection of recombinant HER2 ECD protein. Hybridoma clones were screened by HER2 ECD ELISA assay. Five hybridoma clones (namely BH1, BH2, BH5, BH6 and BH7) were selected for further analyses, based on their reactivity with HER2 ECD, but not EGFR ECD (Additional file 1). All monoclonal antibodies were isotyped as IgG1 with κ-light chains. Antibodies were purified from hybridoma supernatants using Protein G affinity chromatography and used for further studies.

### Antibody reactivities against breast cancer cell lines

**Figure 1:**
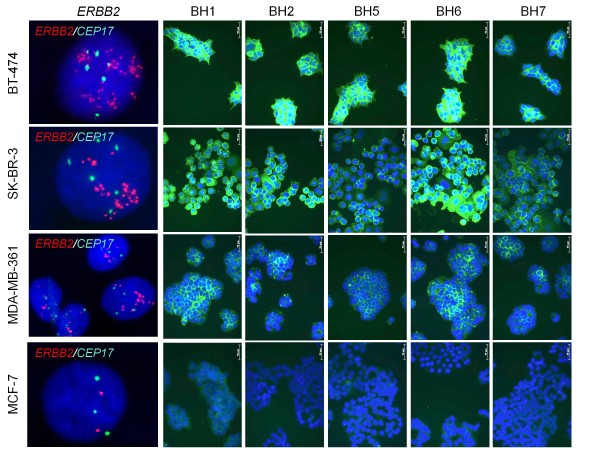
** Strong binding of anti-HER2 antibodies to *ERBB2 *-amplified breast cancer cell lines.** ERRB2 amplification was analyzed by FISH analysis. Cells were harvested at confluence, fixed and subjected to dual-color FISH using Texas Red-labeled *ERBB2* (red) and FITC-labeled chromosome 17 α-satellite CEP17 (green) DNA probes (left panel). For generation of anti-HER2 monoclonal antibodies, mice were immunized with SK-BR-3 cells, followed by a recombinant protein composed of extracellular domain of HER2 fused to human IgG1 Fc domain (HER2 ECD). Antibodies were purified from conditioned hybridoma cell culture media by affinity chromatography. For immunofluorescence assay, cells were seeded on coverslips in 6-well plates, incubated 36 h in cell culture medium, fixed, and permeabilized. Fixed cells were incubated with anti-HER2 antibodies, and the primary antibody binding was detected using Alexa 488- conjugated anti-mouse IgG (BH1, BH2, BH5, BH6, BH7 panels; green). Nuclei were counterstained with DAPI (blue) and pictures were acquired using Zeiss Axio Imager.A1 microscope.

Anti-HER2 antibodies were tested using five breast cancer cell lines with different *ERBB2* copy numbers. Fluorescence in situ hybridization (FISH) using probes against *ERBB2* gene and chromosome 17 centromere showed that MCF-7 had three chromosome 17 and two *ERBB2* signals. MDA-MB-361 provided 4–8 chromosome 17, and 8–12 *ERBB2* signals. Both SK-BR-3 and BT-474 displayed 5–6 chromosome 17 signals and highly amplified *ERBB2* signals (Figure 1, left panel). Most of T47D cells displayed three chromosome 17 and three *ERBB2* copies (data not shown). These findings were in perfect agreement with a previous study reporting 47, 43, 11 and 2.5 copies of the *ERBB2* gene in BT-474, SK-BR-3, MDA-MB-361 and MCF-7, respectively [37]. When tested by indirect immunfluorescence assay, all five anti-HER2 antibodies displayed strong fluorescence intensities with both BT-474 and SK-BR-3 cells. The signals obtained with MDA-MB-361 were positive but not strong, but those provided by MCF-7 were negative or barely detectable (Figure 1, BH1, BH2, BH5, BH6, BH7 panels). 

**Figure 2:**
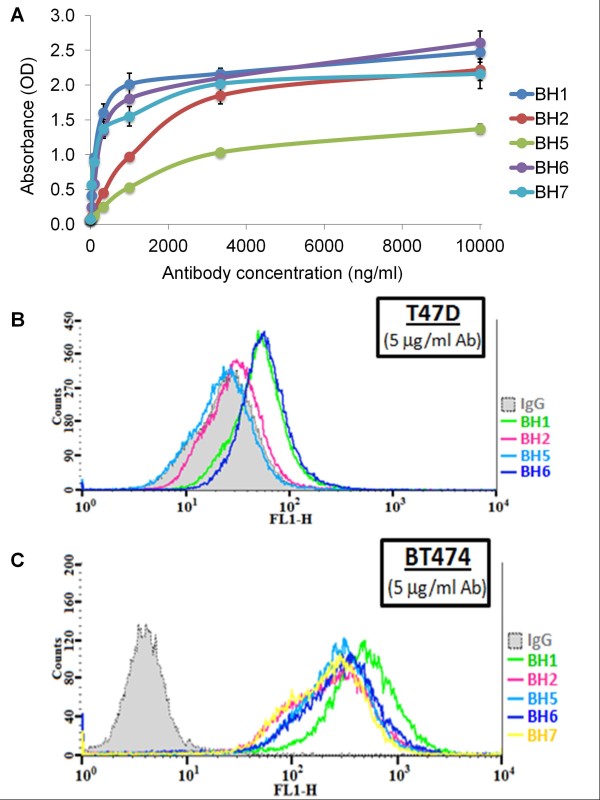
** Binding affinities of anti-HER2 antibodies.** (**A**) HER2 ECD ELISA assay showing the binding strengths of five monoclonal antibodies. HER2 ECD-coated plates were incubated with increasing concentrations of anti-HER2 antibodies, followed by alkaline phosphatase-conjugated anti-mouse IgG antibodies. Y axis shows the absorbance (OD) reading at 405 nm following incubation with alkaline phosphatase substrate. T-bars: SD. (**B**) and (**C**), Flow cytometric analysis of antibody binding to low HER2-expressing T47D (**B**) and high HER2-expressing BT-474 (**C**) breast cancer cells. Fixed single-cell suspensions were incubated with anti-HER2 antibodies, followed by Alexa-488-conjugated secondary antibodies and fluorescence intensities were measured using flow cytometry.

The binding strengths of antibodies were compared using HER2 ECD ELISA and flow cytometry. ELISA test provided evidence of strong binding capacity for BH1, BH6 and BH7 antibodies. BH5 antibody displayed lowest binding capacity, whereas BH2 had intermediate activity. These observations strongly suggested that we produced three anti-HER2 antibodies with high affinity, one with low and one with intermediate affinities (Figure 2A). Flow cytometric analyses provided similar findings. When tested with T47D breast cancer cell line expressing low levels of HER2 [38], BH1 and BH6 displayed highest shifts in staining intensity as compared to isotype control antibody. The binding of BH5 was not detectable, whereas BH2 displayed a weak binding (Figure 2B). However, when tested with BT-474 that has *ERBB2* gene amplification (Figure 1, left panel), all antibodies displayed strong intensity shifts, BH1 producing the best shift (Figure 2C). 

### Newly generated anti-HER2 antibodies recognize conformation-dependent epitopes

Bioactive antibodies that are most effective against HER2-overexpressing tumors recognize conformational epitopes located at ECD subdomains involved in either receptor activation [21] or receptor dimerization [23]. Thus, the bioactivity of anti-HER2 antibodies may be strongly dependent on how they occupy the critical regions on their target. We performed a series of analyses to identify epitopes recognized by newly raised monoclonal antibodies. In these studies, Mouse IgG1 isotype antibody and humanized Trastuzumab were used as negative and positive controls, respectively. NCL-CB11 (CB11 in short) antibody against intracellular domain of HER2 was also used. Initially performed western blot assays using different breast cancer cell line lysates provided positive signals with CB11 antibody, but not with others (data not shown). Based on these findings, we hypothesized that the epitope(s) recognized by our antibodies were unstable under denaturing conditions of SDS-PAGE, although they remained stable after paraformaldehyde fixation used in indirect immunofluorescence and flow cytometry experiments (Figures 1, 2). Next, we tested whether our antibodies were able to immunoprecipitate HER2 from cell lysates made in RIPA buffer. Cell lysates made from T47D cells were subjected to immunoprecipitation with our antibodies and antibody-bound proteins were tested by western blot assay using CB11 antibody. All antibodies tested were able to immunoprecipitate HER2 under these conditions. The signal obtained with BH5 antibody was weak, as expected (Additional file 2). These findings provided evidence that newly generated antibodies recognized conformation-dependent epitope(s) on endogenously expressed HER2 protein.

### Discovery of a new epitope(s) restricted to the intact form of the extracellular domain of HER2

**Figure 3:**
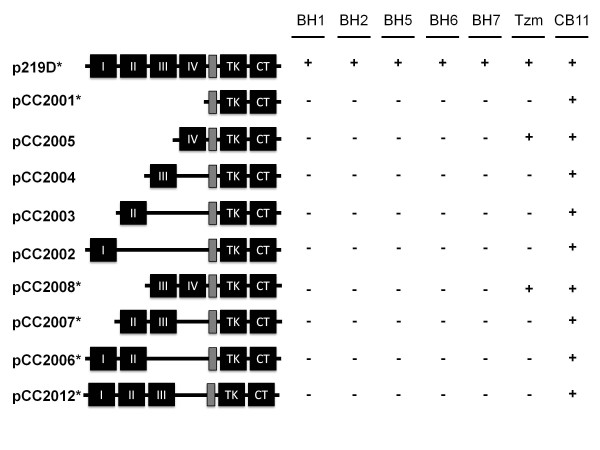
** Schematic representation of antibody epitope mapping studies.** Mammalian expression plasmids expressing full-length HER2 (p219D) or partially deleted HER2 were transfected into Huh7 cells. Anti-HER2 antibody reactivity was tested by indirect immunofluorescence (Figure 4) and immunoprecipitation-western blotting (Additional file 3) techniques. The boxes named as I, II, III, IV represent four subdomains of ECD. Grey box: transmembrane domain, TK: tyrosine kinase domain, CT: carboxyl-terminal domain. Tzm: Trastuzumab.+: antibody reactivity, -: no antibody reactivity. Stars (*) near plasmid names indicate that immunofluorescence data were confirmed with immunoprecipitation-western blot assay.

**Figure 4:**
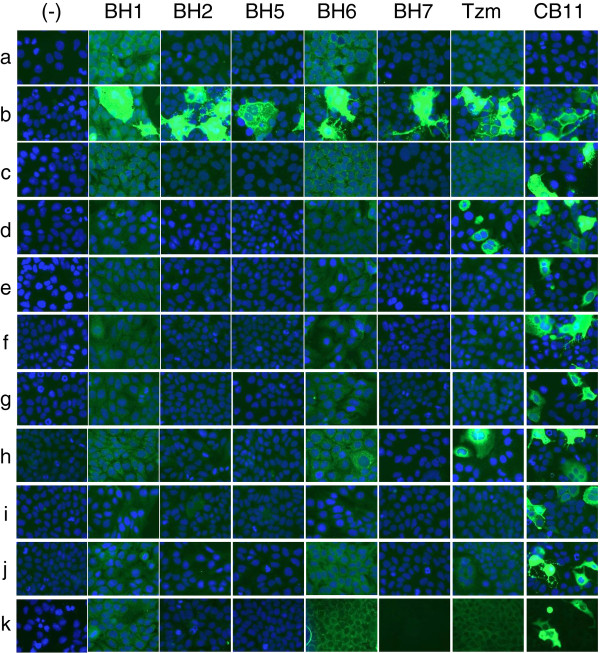
** Epitope mapping of anti-HER2 antibodies using indirect immunofluorescence assay.** Huh7 cells were transfected in 6-well plates using a set of mammalian expression plasmids encoding full-length or N-terminally truncated HER2 protein. Transfected cells were cultivated for 48 h, and subjected to indirect immunofluorescence assay. Transfected cells that were recognized by specific antibodies displayed strong green fluorescence. The letters from (a) to (k) indicate the plasmids in the following order: pDEST26 (empty vector), p219D, pCC2001, pCC2005, pCC2004, pCC2003, pCC2002, pCC2008, pCC2007, pCC2006, pCC2012 (see Figure 3 for description of HER2-expression plasmids). (−) No primary antibody, Tzm: Trastuzumab, CB11: a monoclonal antibody recognizing an epitope at intracellular domain of HER2 that was used as a positive control for expression of transfected plasmids.

In order to delineate the epitope(s) recognized by the antibodies, we constructed a series of plasmids to express truncated forms of HER2 at its ECD (Figure 3). Huh-7 cells that express low levels of endogenous HER2 protein [39] were transiently transfected with plasmids to express different forms of HER2, and immunoreactivity of each antibody was tested by indirect immunofluorescence and western blotting following immunoprecipitation. In immunofluorescence assay, CB11 antibody that was used as a positive control for expression of full length and truncated forms of HER2 transgene identified strongly positive transfected cells with all plasmids tested (Figure 4, right panel). Another control was Trastuzumab that is directed against an epitope located at domain IV of the extracellular region of HER2 [21]. All truncated forms of HER2 protein containing domain IV provided strongly positive signals with Trastuzumab (Figure 4, Tzm panel). Surprisingly, all five of our antibodies recognized only full-length HER2. They did not react with recombinant HER2 protein forms lacking any one of the ECD subdomains (Figure 4, panels BH1, BH2, BH5, BH6, BH7). Immunoprecipitation-western blotting assays following transient expression of full length and four truncated forms of HER2 confirmed immunofluorescence findings (Additional file 3). We deduced that newly generated antibodies are directed to an epitope, or closely related epitopes present only on structurally intact ECD of HER2. In other words, the reactivity of antibodies was lost when any of the four subdomains of the HER2 ECD was deleted. Thus, all five antibodies described in the report required the structural integrity of ECD for recognition of HER2 receptor (Figure 3). 

### Effect of anti-HER2 antibodies on cell growth

**Figure 5:**
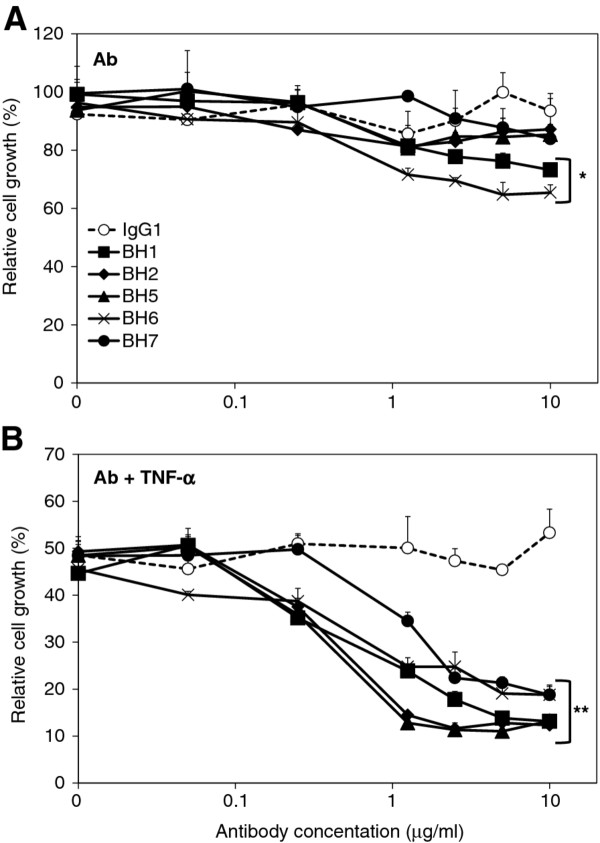
** Anti-HER2 antibodies exhibit enhanced anti-growth effect in the presence of tumor necrosis factor-α in SK-BR-3 cells.** (**A**) SK-BR-3 cells were plated onto 96-well plates and incubated at 37°C with 5% CO_2_. After 2 h of incubation, antibodies (0–10 μg/ml) were added, and cells were incubated for 6 days. (**B**) Antibodies (0–10 μg/ml) were added as described in (**A**), and cells were incubated for 4 h prior to TNF-α (1000 U/ml) addition to the culture medium. Cells amount was determined by Sulforhodamine B assay after 6 days of treatment. T. Bars, SD; * p < 0.006, ** p < 0.0005, (**A**, **B**).

The bioactivity of antibodies was first tested against SK-BR-3 cells. Affinity purified anti-HER2 antibodies were added at increasing concentrations into the culture media and relative cell growth was tested following 6 days of cell culture. Only BH6 and BH1 antibodies displayed modest but significant inhibitory effect on target cells, by lowering relative cell growth to 65% and 73% respectively, as compared to isotype control antibody (Figure 5A; p < 0. 006 at 10 μg/ml antibody concentration). In contrast, BH2, BH5 and BH7 antibodies did not affect the growth of SK-BR-3 cells.

### Effects of anti-HER2 antibodies in combination with tumor necrosis factor-α

It has been previously reported that amplified expression of HER2 in breast cancer cells induces resistance to TNF-α cytotoxicity [40,41] that can be sensitized by Trastuzumab [42]. Therefore, we asked whether our anti-HER2 antibodies were able to sensitize SK-BR-3 cells to TNF-α. We performed antibody treatment experiments in the presence of 1000 U/ml of TNF-α (Figure 5B). In the absence of antibody, TNF-α alone induced nearly 50% growth inhibition. This effect was not modified significantly in the presence of increasing concentrations of IgG1 isotype control antibody (up to 10 μg/ml tested). In sharp contrast, all five anti-HER2 antibodies strongly enhanced TNF-α-induced growth inhibition (p < 0.0005 at 10 μg/ml antibody concentration). As little as 0.25 μg/ml anti-HER2 antibody was sufficient to significantly enhance TNF-α-mediated growth inhibition, as compared to IgG1 isotype control antibody at the same concentration (p < 0.005). Only one antibody, namely BH7, displayed a slightly weaker enhancer activity, reaching significance at 1.25 μg/ml concentration (p <0.02). Enhancer effects of anti-HER2 antibodies were nearly saturated at 2.5 μg/ml concentration, reaching to 80-90% growth inhibition depending on antibody (Figure 5B).

### Cell-dependent interactions between anti-HER2 antibodies and tumor necrosis factor-α creating both synergistic and antagonistic growth effects

**Figure 6:**
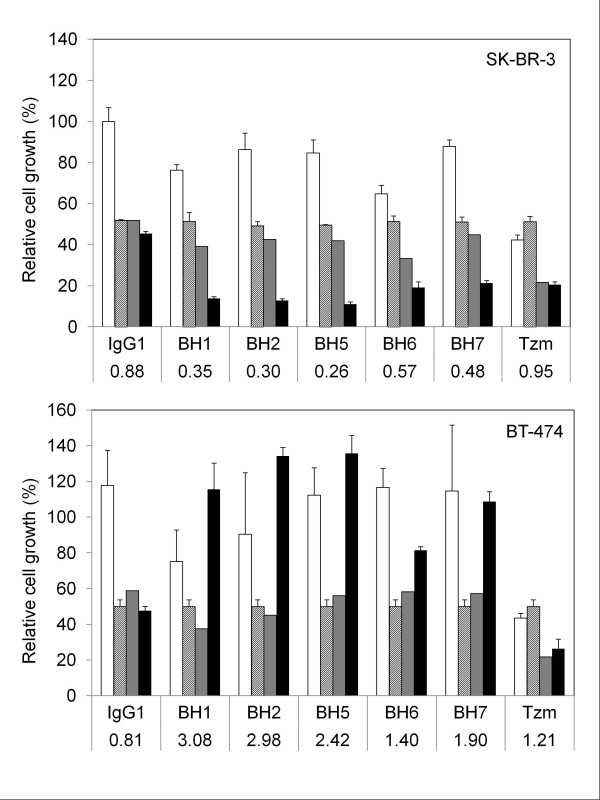
** Synergy and antagonism between anti-HER2 antibodies and tumor necrosis factor-α.** Anti-Her2 antibodies and TNF-α acted synergistically in SK-BR-3 cells (top), but antagonistically in BT-474 cells (bottom). Growth effects observed under antibody (white columns), TNF-α (striped columns), and antibody + 1000 TNF-α (black columns) were obtained as follows. Cells were plated onto 96-well plates and incubated at 37°C with 5% CO_2_. After 2 h of pre-incubation, antibodies (5 μg/ml) were added, and cells were incubated for 6 days to study the effects of antibodies alone (white columns). The effects TNF-α (1000 U/ml) alone (striped columns) were studied by adding this cytokine into cell culture medium after 4 h of pre-incubation. For combined treatments, antibodies (5 μg/ml) and TNF-α (1000 U/ml) were added after 2 h and 4 h of pre-incubations, respectively (black columns). Cells amount was determined by Sulforhodamine B assay after 6 days of treatment. Growth level under a drug condition was defined as the growth under that condition normalized by growth under no drug condition. Expected growth level under no interaction (gray columns) was calculated by multiplying the growth levels under each individual drug. The observed growth level for each combination was divided by the expected growth level to find an interaction score according to Bliss Independence Model for drug interactions (displayed under each antibody label). The combination of TNF-α with isotype control antibody (IgG1) or Trastuzumab (Tzm) provided scores near 1, meaning no interaction or independence in both cell lines. New anti-HER2 antibodies provided scores less than 0.57 in SK-BR-3 cells, meaning synergy. In contrast their interaction scores were more than 1.40 in BT-474 cells, meaning antagonism.

Based on observations with SK-BR-3 cells, we analyzed interactions between anti-HER2 antibodies and TNF-α, using five different breast cancer cell lines. We compared growth responses to (i) 5 μg/ml of each individual antibody, (ii) 1000 U/ml of TNF-α, and (iii) 5 μg/ml antibody and 1000 U/ml of TNF-α in combination. An interaction score for each combination was calculated as described in the Methods, where a score of 1 means no interaction, or independence; a score smaller than 1 means synergy, and a score larger than 1 means antagonism [32]. In Figure 6, we analyzed TNF-α and anti-HER2 antibody interactions as compared to IgG1 isotype control antibody. IgG1 control antibody interactions with TNF-α provided scores between 0.81-0.88. Therefore, we considered values between 0.8-1.2 as an indication of non-interaction. When tested with SK-BR-3 cell line, interaction scores obtained for TNF-α and new anti-HER2 antibody combinations ranged between 0.26 and 0.48 (Figure 6, top). Synergy scores obtained with BH1, BH2 and BH5 were particularly strong (scores: 0.35, 0.30 and 0.26, respectively). In contrast, there was no interaction between Trastuzumab and TNF-α (score = 0.95). Thus, all five new anti-HER2 antibodies acted synergistically with TNF-α to inhibit the growth of SK-BR-3 cells. 

Further studies indicated that the interaction between anti-HER2 antibodies and TNF-α was cell-dependent. Indeed, we observed a completely opposite response with another breast cancer cell line, namely BT-474 (Figure 6, bottom). This cell line responded to a few anti-HER2 antibodies modestly albeit significantly (BH1, p < 0.05; Tzm, p < 0.003). The response to TNF-α alone was about 50% growth inhibition, as observed with SK-BR-3 cells. Novel anti-HER2 antibodies abolished TNF-α-induced growth inhibition either completely (BH1, BH2, BH5, BH7) or partially (BH6) in BT-474 cells. Interactions scores of novel anti-HER2 antibodies ranged between 1.40 and 3.08, all indicative of an antagonistic interaction with TNF-α. Again, Trastuzumab had no major interaction with TNF-α (score = 1.21). Data on interaction analyses performed with MDA-MB-361, MCF-7 and T47D were shown in Additional file 4. When tested alone with MDA-MB-361 cells, some antibodies (BH1, BH2, BH7) displayed significant growth inhibition (>50% inhibition, p < 0.0003). Trastuzumab had no detectable anti-growth effect, but BH5 and BH6 displayed only modest activity (27% and 35% growth inhibition, respectively; p < 0.02). TNF-α was highly toxic to this cell line causing >95% growth inhibition. Antibody co-treatments rescued TNF-α induced growth inhibition only slightly. Thus, drug interaction analysis was not conclusive. The growth of MCF-7 cells did not appear to be affected by antibody treatment. In contrast, these cells were the most sensitive ones to TNF-α with nearly 100% growth inhibition. Antibody co-treatments did not change their response to TNF-α. T47D cells were resistant to both anti-HER2 antibody and TNF-α treatments, either alone or in combination.

### Detailed analysis of interaction between BH1 antibody and tumor necrosis factor-α

**Figure 7:**
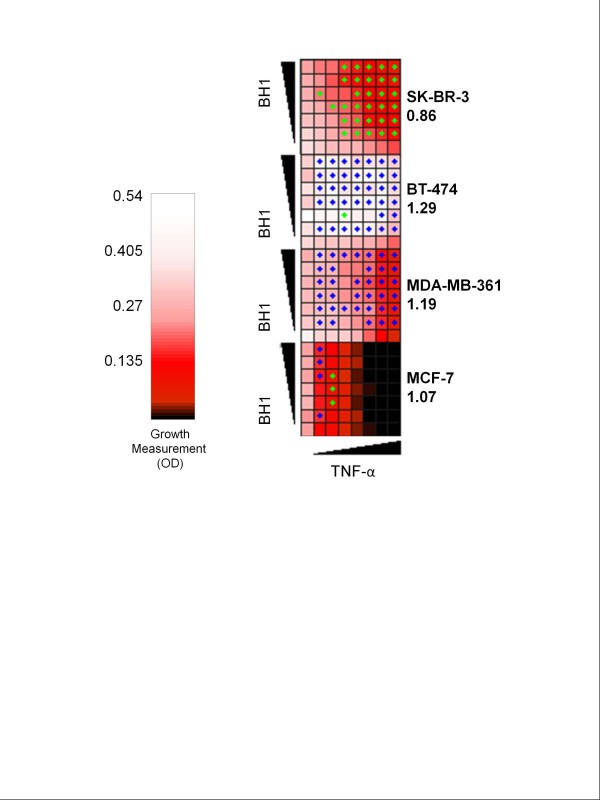
** Anti-HER2 antibody and tumor necrosis factor-α interaction is cell type-specific.** The growth of breast cancer cell lines under various combinations of BH1 antibody (0, 0.161, 0.312, 0.625, 1.25, 2.5, 5.0 μg/ml) and TNF-α (0, 8, 16, 32, 62.5, 125, 250, 500 U/ml) treatments was experimentally tested in triplicate and the average relative growth levels were calculated. Heatmap represents the cell growth (as measured by OD) under different conditions. Synergistic and antagonistic interactions are indicated by green and blue dots, respectively. Numbers associated with cell line names indicate interaction scores. According to this checkerboard assay, BH1 and TNF-α show synergy in SK-BR-3 cells, but antagonism in BT-474 and MDA-MB-361 cells. There is no interaction in MCF-7 cells.

Drug interaction analyses described above were performed using fixed amounts of antibodies and TNF-α. We obtained clear-cut results with only SK-BR-3 and BT-474 cells. Therefore, we decided to test the effects of combined treatment of anti-HER2 antibody BH1 and TNF-α on four breast cancer cell lines using a checkerboard assay. BH1 was selected as a representative of newly generated anti-HER2 antibodies. Each cell line was grown on a 7×8 grid of BH1 antibody and TNF-α combinations. The concentration of TNF-α was increased on the x-axis (0, 8, 16, 32, 62.5, 125, 250 and 500 U/ml) and the concentration of BH1 antibody was increased on the y axis (0, 0.16, 0.312, 0.625, 1.25, 2.5 and 5 μg/ml). Such an experimental setting allowed us to measure the growth of each cell type in 42 concentration combinations of BH1 antibody and TNF-α, in addition to the growth of cells under each ‘drug’ alone. This experiment was done in triplicate for each cell type. The average growth measures are shown in Figure 7. We analyzed the growth measures using the same definition of drug synergy based on Bliss Independence Model as described above. We found concentration combinations where BH1 and TNF-α are significantly synergistic or antagonistic, which are indicated as green or blue dots on Figure 7, respectively. For each of the drug interaction experiments, we next computed a single interaction score as described previously [34]. These scores, which are interpreted as described above, are shown in Figure 7. According to this, BH1 and TNF-α exhibited synergy in SK-BR-3 cells, while they were antagonistic in BT-474 and MDA-MB-361 cell types, when all concentration combinations are considered. Interestingly, BH1 antibody, even at a very low dose (0.16 μg/ml), strongly suppressed the growth inhibition triggered by TNF-α in BT-474 cells. These observations confirm strong synergistic and antagonistic interactions of TNF-α and anti-HER2 antibodies in SK-BR-3 and BT-474, respectively (Figure 6). The interaction score for MCF-7 was very close to 1, suggesting independence. 

### Mechanisms of growth inhibition by anti-HER2 antibodies and tumor necrosis factor-α

**Figure 8:**
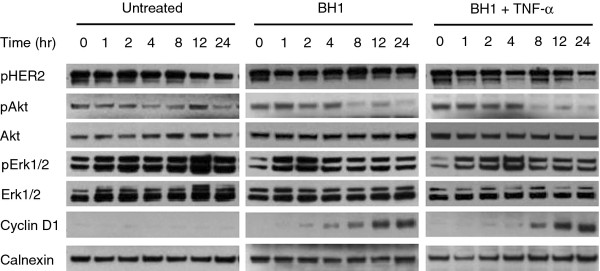
*** In vitro *molecular responses of SK-BR-3 cell to anti-HER2 antibody BH1 and tumor necrosis factor-α. ** SK-BR-3 cells were treated up to 24 h with BH1 antibody (10 μg/ml), TNF-α (1000 U/ml) or the combination of both agents. Cell lysates were prepared from cells harvested at indicated times (hr, hours) and the expressions of phospho-HER2 (pHER2), phospho-Akt (pAkt), total Akt (Akt), phospho-ERK1/phospho-Erk2 (pErk1/2), total Erk1/Erk2 (Erk 1/2) and Cyclin D1 were analyzed by western blotting. Calnexin was used as a loading control. Untreated cells were incubated in a parallel experiment in the absence of any treatment.

In cancer cells, overexpressed HER2 is believed to be constitutively active as homodimers and/or as heterodimers with ligand-dependent HER3 [6]. Downstream signaling from HER2 involves PI3K-dependent activation of Akt and Ras-dependent activation of ERK1/ERK2 proteins by phosphorylation, mediating cell survival and proliferation activities of HER2 [6]. Therapeutically relevant Trastuzumab and Pertuzumab antibodies inhibit both Akt and ERK1/ERK2 phosphorylation, leading to growth inhibition [24,43-45]. TNF-α signaling generates both cell survival and apoptosis signals, depending on the activation of NFκB transcription factor [46]. We further investigated the mechanisms of growth inhibition in responsive SK-BR-3 cells. As shown in Figure 8 left panel, untreated SK-BR-3 cells displayed relatively stable expression of phospho-HER2, phospho-Akt, total Akt, phospho-Erk1/2 and total Erk1/2. Cyclin D1 levels were undetectable under this experimental condition. Cells treated with 10 μg/ml BH1 antibody inhibited Akt phosphorylation that was clearly detectable between 8 h and 24 h of treatment. There was also a partial inhibition of Erk1/2 phosphorylation between 4 h and 24 h of treatment. In parallel with these changes, we also observed progressive accumulation of cyclin D1 levels detected as early as 4 h of treatment. In contrast, HER2 phosphorylation was not affected by antibody treatment (Figure 8 middle panel). Cells co-treated with 10 μg/ml BH1 and 1000 U/ml TNF-α displayed essentially the same type of alterations (Figure 8 right panel). Thus, TNF-α appeared not to modify the effects of the anti-HER2 antibody on phospho-Akt, phospho-ERK1/2 and Cyclin D1 in SK-BR-3 cell line. Indeed, treatment of cells with 1000 U/ml TNF-α alone did not affect any of the parameters studied, except a delayed inhibition of Akt phosphorylation at 24 h (Additional file 5). 

**Figure 9:**
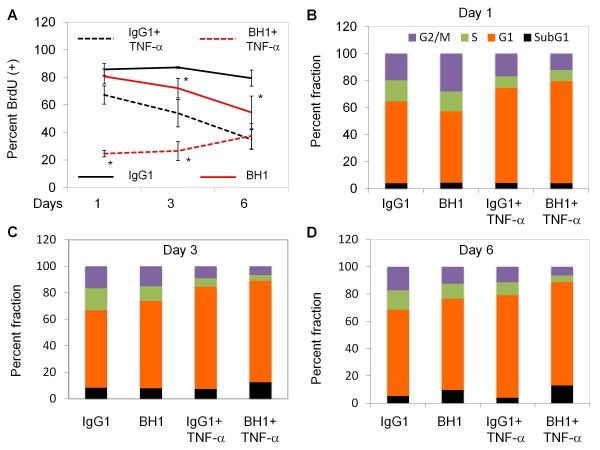
** Effects of anti-HER2 antibody BH1 and tumor necrosis factor-α on DNA synthesis and cell cycle in SK-BR-3 cells.** (**A**) Effects on DNA synthesis were tested by 24-h BrdU incorporation assay. Cells were plated on coverslips in 12-well plates, treated as described in Figure 5, and were exposed to BrdU during 24 h prior to indicated days. Nuclear BrdU was detected by indirect immunofluorescence assay. Nuclei were counterstained with DAPI, examined using Zeiss Axio Imager.A1 microscope. Representative photographs (3–5 frames per coverslip) were acquired. Percent BrdU incorporation was calculated after manual counting of BrdU^+^ and BrdU^-^ nuclei. * Denotes p-values ≤ 0.05 (BH1 *vs.* IgG1, BH1 + TNF-α *vs.*IgG1 + TNF-α). T-bars: SD. (**B**-**D**). Effects on cell cycle were analyzed by flow cytometry. SK-BR-3 cells were treated with 20 μg/ml BH1 or IgG isotype control antibody in the presence or absence of 1000 U/ml TNF-α. Cells were fixed on day 1 (**B**), day 3 (**C**) and day 6 (**D**), stained with propidium iodide and the cell cycle distribution was analysed with flow cytometry. Fraction of cells present in G2/M-phase (G2/M), S-phase (S) and G1-phase (G1) are shown in purple, green and orange respectively. Fraction of apoptotic cells observed as a SubG1 peak is shown in black.

Cyclin D1 accumulates in G1 and G2 phases of cycling cells with a loss of expression in S phase cells [47]. Accordingly, Cyclin D1 accumulation could indicate progressive accumulation of cells at G1 and G2/M phases under anti-HER2 antibody treatment. We tested this hypothesis by exposing BH1 and/or TNF-α-treated cells to BrdU labeling and cell cycle analysis experiments during 6 days of treatment. Tests were performed at days 1, 3 and 6 (Figure 9A). SK-BR-3 cells have doubling times varying between 15 h and 36 h [48,49]. In order to detect the ratio of cells at G1/G0 phase, we used a modified DNA labeling protocol by exposing cells to BrdU for 24 h prior to each experimental time. As compared to IgG1 isotype control antibody, BH1 induced a significant decrease in the number of BrdU^+^ cells at days 3 and 6 (p ≤ 0.05). Treatment of cells with IgG1 and TNF-α resulted in a more pronounced decrease of BrdU^+^ cells that dropped progressively to 35% at day 6. When compared to IgG1 + TNF-α-treated cells, BH1 + TNF-α-treated cells responded immediately by a radical decrease of BrdU incorporation rates to 25% and 26% at days 1 and 3, respectively (p ≤ 0.05). However, this inhibition was not stable and reversed to 37% at day 6 (Figure 9A). These findings indicated that both BH1 and TNF-α significantly increased the number of cells at G1/G0 phase, although this effect was delayed in BH1-only treatment. Sharp and immediate decrease in BrdU (+) cells with BH1 + TNF-α co-treatment confirmed the synergy between BH1 and TNF-α in these cells. Cell cycle analyses confirmed these findings (Figure 9B-D). As compared to IgG1 isotype control antibody-treated cells, BH1-treated cells first showed an increase in G2 cells at day 1, followed by a progressive increase of G1 cells at days 3 and 6. This was accompanied with a decrease in S phase cells. TNF-α treatment resulted in an immediate and stable response with an increase in G1 cells, concomitant with a decrease of S phase cells at all time points tested. BH1 + TNF-α treatment exaggerated this response, leading to a stronger accumulation of G1 phase cells, associated with a depletion of G2 phase cells. We concluded that both BH1 antibody and TNF-α were able to induce sustained accumulation of SK-BR-3 cells at G1/G0 phase, and their combination amplified this response particularly in early times of co-treatment. 

## Discussion

This report describes five novel anti-HER2 monoclonal antibodies that have been produced by immunizing mice with HER2-overexpressing whole cells, followed by a boost with recombinant HER2 ECD homodimer protein. Immunoprecipitation and western immunoblotting studies indicated that all five antibodies were directed against conformation-dependent epitope(s) present on the endogenous HER2 protein. Epitope mapping with recombinant HER2 protein forms that have been truncated at ECD domain indicated that all five antibodies react only with the native form of cellular HER2, being unable to recognize truncated proteins lacking any of the four subdomains. Although we do not yet know whether our antibodies recognize identical, overlapping or independent epitopes on the intact ECD domain, it is evident that their epitope(s) is different from those recognized by Trastuzumab and Pertuzumab. Trastuzumab, a therapeutic antibody already approved for clinical use, recognizes an epitope restricted to subdomain IV, involved in receptor activation [21]. Pertuzumab, a therapeutic antibody tested in different clinical trials, recognizes a distinct epitope located on subdomain II, involved in receptor dimerization [23]. Antibodies directed to ECD epitope(s) different from other anti-HER2 therapeutic antibodies may serve as new analytical tools to study the structural organization of HER2 at the cell surface that may be present as monomers, homodimers and heterodimers depending on cell type and extracellular environment such as the availability of ligands that activate heterodimer partners of HER2. Dynamic changes at the receptor ECD in relation with transcriptional, translational and post-translational modifications can be monitored with these antibodies, both biochemically and biologically.

*In vitro* effects of novel anti-HER2 antibodies were tested against five different breast cancer cell lines displaying different degrees of *ERBB2* amplification and HER2 expression. Individually tested novel anti-HER2 antibodies were weakly effective as growth inhibitors (Figure 6). This was not surprising. Indeed, Trastuzumab (4D5) has a modest growth inhibitory effect (56%), and Pertuzumab (2C4), the best therapeutic alternative to Trastuzumab, induces only 20% growth inhibition [42]. Based on the synergy between Trastuzumab and Pertuzumab directed to different epitopes of HER2 [24], we performed studies to test combinatory anti-growth effects of our anti-HER2 antibodies with Trastuzumab on SK-BR-3 cells. Preliminary data suggested a synergy between them, raising the possibility that our antibodies may be combined with Trastuzumab or Pertuzumab to enhance anti-cancer activity. This will be the focus of further work.

The most striking feature of novel anti-HER2 antibodies was their differential interaction with TNF-α depending on cell type. They synergized with TNF-α on SK-BR-3 cells by raising growth inhibition from 50% to nearly 90%. In sharp contrast, the same antibodies displayed strong antagonism with TNF-α on BT-474 cells and MDA-MB-361 cells by abolishing TNF-α-induced growth inhibition either completely (BT-474 cells) or partially (MDA-MB-361 cells). Trastuzumab did not have any synergistic or antagonistic effects on TNF-α-mediated growth inhibition in these cell lines (Figure 6). The sensitization of SK-BR-3 cells to TNF-α cytotoxicity by mouse monoclonal antibody 4D5 (from which Trastuzumab has been humanized) has been reported [42], but there is no previous data on synergy or antagonism between anti-HER2 antibodies and TNF-α, to our best knowledge.

TNF-α is one of the most potent anti-tumor factors when used at high concentrations, but its systematic toxicity hampers its clinical applications [50]. The sensitization of SK-BR-3 cells to TNF-α with anti-HER2 antibodies provides experimental evidence for combination of low TNF-α doses with anti-HER2 antibody treatment to achieve effective anti-growth effects. Based on checkerboard analyses reported in Figure 7, a synergistic anti-growth response could be obtained from SK-BR-3 cells with as low as 32 U/ml TNF-α under *in vitro* conditions. But, one caveat is that the same antibody-TNF-α combination could produce completely opposite responses in other cells, as reported here for BT-474 and MDA-MB-361 cells. For example, as little as 0.1 mg/ml BH1 antibody was able to abolish TNF-α cytotoxicity in BT-474 cells.

The mechanisms of differential responses of breast cancer cell lines to combined treatment with anti-HER2 antibody and TNF-α merit further investigation. Here, we investigated the mechanisms of growth response of SK-BR-3 cells to combined treatment. The analysis of several downstream targets of HER2 signaling indicated that BH1 antibody did not inhibit HER2 phosphorylation, yet it was able to inhibit the phosphorylation of Akt and ERK1/2 kinases. These changes resulted in a progressive increase of cyclin D1 protein levels. DNA synthesis was inhibited and cells accumulated progressively at G1 phase of the cell cycle. The addition of TNF-α to BH1 treatment dramatically enhanced these responses leading to an immediate and stable growth arrest.

## Conclusions

Newly generated anti-HER2 monoclonal antibodies allowed the identification of a novel epitope on HER2 receptor that is restricted to the intact form of its ECD. These antibodies displayed cell type-dependent interactions with TNF-α, resulting in synergistic, antagonistic or independent anti-growth effects. The synergistic activity was associated with inhibition of Akt and ERK1/2 phosphorylation leading to immediate and stable cell cycle arrest.

## Abbreviations

AP: Alkaline phosphatase; BrdU: Bromodeoxyuridine; DAPI: 4',6-diamidino-2-phenylindole; DMEM: Dulbecco’s modified eagle medium; ECD: Extracellular domain; EDTA: Ethylene diamine tetra-acetic acid; EGFR: Epidermal growth factor receptor; ELISA: Enzyme linked immunosorbent assay; ErbB2: Epidermal growth factor receptor 2; FCS: Fetal calf serum; FITC: Fluorescein isothiocyanate; FPLC: Fast protein liquid chromatography; HER2: Human epidermal growth factor receptor 2; HRP: Horse radish peroxidase; IgG1: Immunoglobulin G1 isotype; MUC4: Mucin4; PI3K: Phosphotidylinositol-3-kinase; OD: Optical density; PAGE: Polyacrylamide gel electrophoresis; PNPP: *p*-nitrophenyl phosphate disodium salt; P/S: Penicillin/streptomycin; PVDF: Polivinilidene fluoride; RIPA: Radioimmunoprecipitation assay; SD: Standard deviation; SDS: Sodium dodecyl sulphate; SFM: Serum free media; SRB: Sulforhodamine B; TBS-T: Tris-buffered saline + Tween-20; TCA: Trichloroacetic acid; TNF-α: Tumor necrosis factor- α; Tzm: Trastuzumab.

## Competing interests

The authors declare that they have no competing interests.

## Authors’ contributions

TY and MO designed the study. CC designed, performed and analyzed all experiments except FISH analysis. IT and CC performed checkerboard growth assays. MC analyzed growth effects by drug interaction modeling. SC performed FISH analyses. MO, TY and CC wrote the manuscript. All authors read and approved the final manuscript.

## Pre-publication history

The pre-publication history for this paper can be accessed here:

http://www.biomedcentral.com/1471-2407/12/450/prepub

## Supplementary Material

Additional file 1 ** Anti-HER2 antibodies did not react with EGFR.** Mice were immunized with SK-BR-3 cells, followed by a recombinant protein composed of extracellular domain of HER2 fused to human IgG1 Fc domain (HER2 ECD). ELISA plates that have been coated with either HER2 ECD or EGFR ECD were incubated with antibody-containing hybridoma supernatants, followed by alkaline phosphatase-conjugated anti-mouse IgG antibodies. The Y axis shows the absorbance (OD) reading at 405 nm following incubation with alkaline phosphatase substrate (assays in duplicate). Click here for file

Additional file 2 ** Antibody binding to endogenously expressed HER2 protein as tested by immunoprecipitation-western blot assay.** T47D-derived lysates were subjected to immunoprecipitation with different anti-HER2 antibodies. Antigen-antibody complexes were captured onto Protein G-conjugated beads, eluted and subjected to western blot assay using CB11 antibody. Blots were overexposed to visualize the weakly positive HER2 immunoprecipitated by BH5 antibody. Tzm: Trastuzumab used as a positive control antibody. (−): No primary antibody. Click here for file

Additional file 3 ** Epitope mapping of anti-HER2 antibodies using combined immunoprecipitation-western blot assay.** Huh7 cells were transfected in 6-well plates using a set of mammalian expression plasmids encoding full-length or N-terminally truncated HER2 protein. Transfected cells were cultivated for 48 h; cell lysates were subjected to immunoprecipitation with different anti-HER2 antibodies. Antigen-antibody complexes were captured onto Protein G-conjugated beads, eluted and subjected to western blot assay using CB11 antibody. Arrows: immunoreactive bands specific for full-length and truncated HER2 protein forms. IP: immunoprecipitation; *: non-specific bands originating from antibodies used for immunoprecipitation. Click here for file

Additional file 4 ** Interaction between anti-HER2 antibodies and tumor necrosis factor-α.** MDA-MB-361 cells were partially sensitive to anti-HER2 antibodies, but highly sensitive to TNF-α (top). MCF-7 cells were resistant to anti-HER2 antibodies, but highly sensitive to TNF-α (middle). T47D cells were resistant to both anti-HER2 antibodies and TNF-α (bottom). Growth measurements observed under 5μg/ml antibody (white columns), 1000 U/ml TNF-α (striped columns), and 5 μg/ml antibody + 1000 U/ml TNF-α (black columns) were obtained experimentally, as described in Figure 6. Growth level under a drug condition was defined as the growth under that condition normalized by growth under no drug condition. Expected growth level under no interaction (gray columns) was calculated by multiplying the growth levels under each individual drug. The observed growth level for each combination was divided by the expected growth level to find an interaction score according to Bliss Independence Model for drug interactions. Interaction scores were not calculated for these cell lines. Click here for file

Additional file 5 ** In vitro molecular responses of SK-BR-3 cells to tumor necrosis factor-α.** SK-BR-3 cells were treated up to 24 h with TNF-α (1000 U/ml). Cell lysates were prepared from cells harvested at indicated times (hr, hours) and the expressions of phospho-HER2 (pHER2), phospho-Akt (pAkt), total Akt (Akt), phospho-ERK1/phospho-Erk2 (pErk1/2), total Erk1/Erk2 (Erk 1/2) and Cyclin D1 were analyzed by western blotting. Calnexin was used as a loading control. Click here for file
