# Supplementary figures and images for: Novel anti-HER2 monoclonal antibodies: synergy and antagonism with tumor necrosis factor-α
Source: BMC Cancer. 2012 Oct 4;12:450. doi: 10.1186/1471-2407-12-450 (PMC3517359; doi:10.1186/1471-2407-12-450)

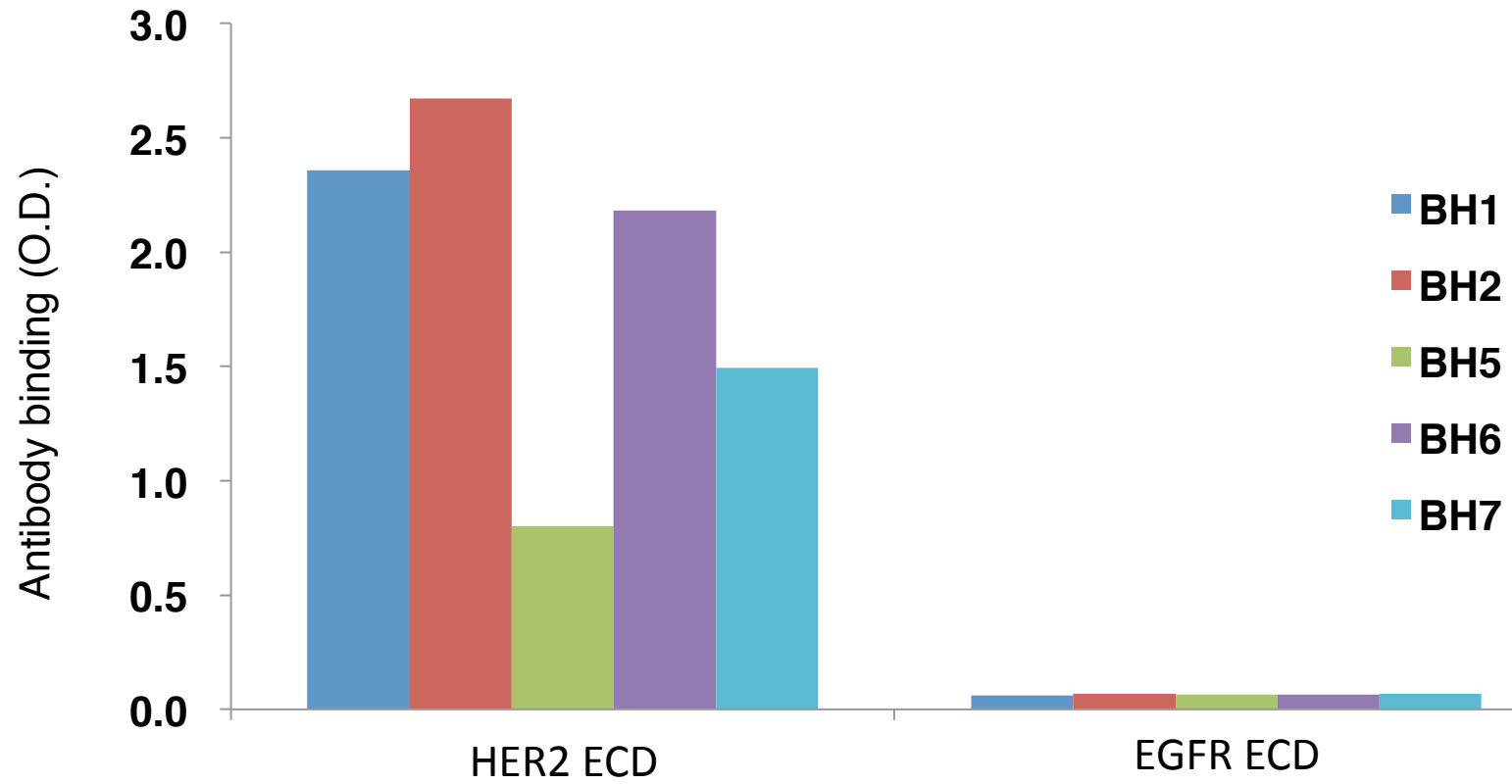

Supplement: Additional file 1 — Anti-HER2 antibodies did not react with EGFR. Mice were immunized with SK-BR-3 cells, followed by a recombinant protein composed of extracellular domain of HER2 fused to human IgG1 Fc domain (HER2 ECD). ELISA plates that have been coated with either HER2 ECD or EGFR ECD were incubated with antibody-containing hybridoma supernatants, followed by alkaline phosphatase-conjugated anti-mouse IgG antibodies. The Y axis shows the absorbance (OD) reading at 405 nm following incubation with alkaline phosphatase substrate (assays in duplicate). [file 1471-2407-12-450-S1.pdf]

IP: BH1 BH2 BH5 BH6 BH7 Tzm (-)

WB: CB11 →

kDa

250 —

170 —

130 —

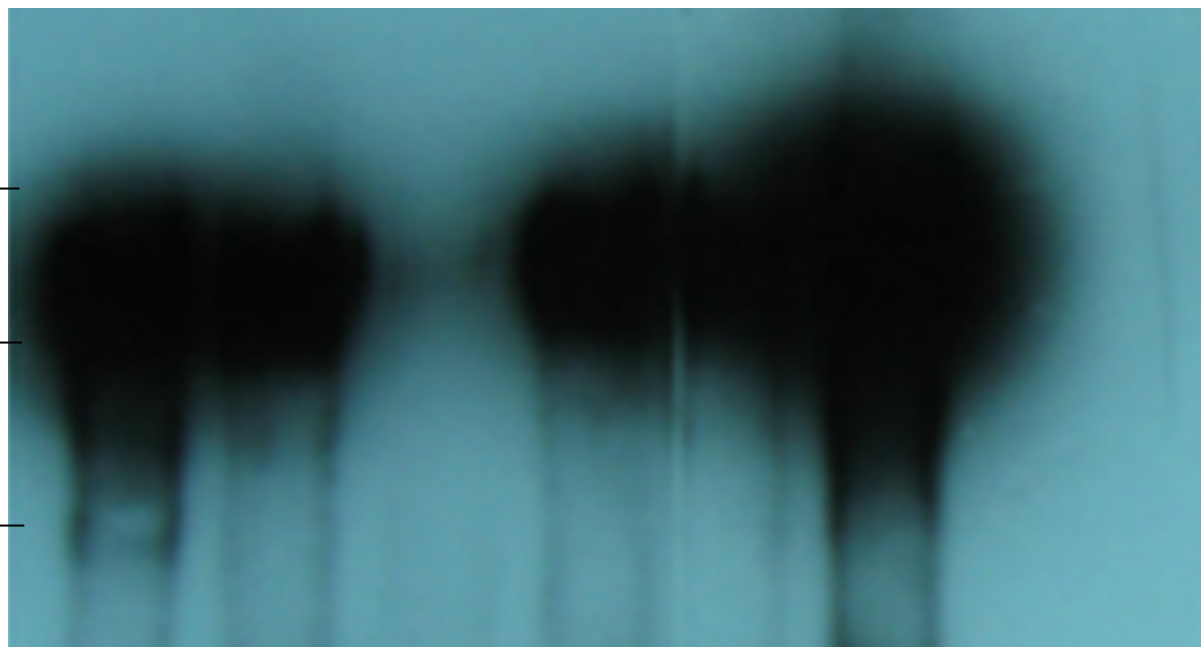

Supplement: Additional file 2 — Antibody binding to endogenously expressed HER2 protein as tested by immunoprecipitation-western blot assay. T47D-derived lysates were subjected to immunoprecipitation with different anti-HER2 antibodies. Antigen-antibody complexes were captured onto Protein G-conjugated beads, eluted and subjected to western blot assay using CB11 antibody. Blots were overexposed to visualize the weakly positive HER2 immunoprecipitated by BH5 antibody. Tzm: Trastuzumab used as a positive control antibody. (−): No primary antibody. [file 1471-2407-12-450-S2.pdf]

# IP

p219D

pCC2001

pCC2006

pCC2007

pCC2008

pCC2012

(-) IgG BH1 BH2 BH5 BH6 BH7 Tzm CB11 Lysate

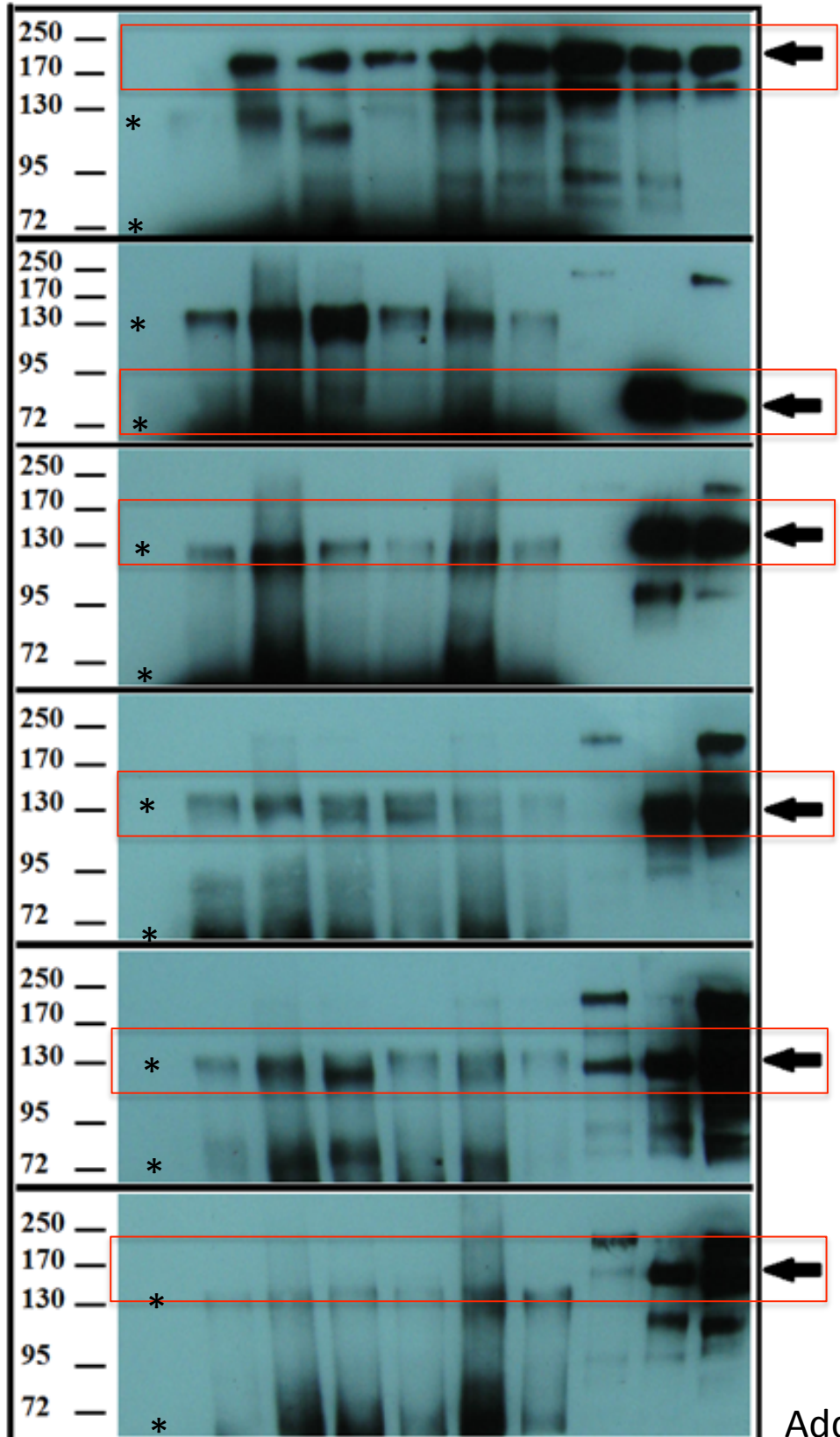

Add. F3

Supplement: Additional file 3 — Epitope mapping of anti-HER2 antibodies using combined immunoprecipitation-western blot assay. Huh7 cells were transfected in 6-well plates using a set of mammalian expression plasmids encoding full-length or N-terminally truncated HER2 protein. Transfected cells were cultivated for 48 h; cell lysates were subjected to immunoprecipitation with different anti-HER2 antibodies. Antigen-antibody complexes were captured onto Protein G-conjugated beads, eluted and subjected to western blot assay using CB11 antibody. Arrows: immunoreactive bands specific for full-length and truncated HER2 protein forms. IP: immunoprecipitation; *: non-specific bands originating from antibodies used for immunoprecipitation. [file 1471-2407-12-450-S3.pdf]

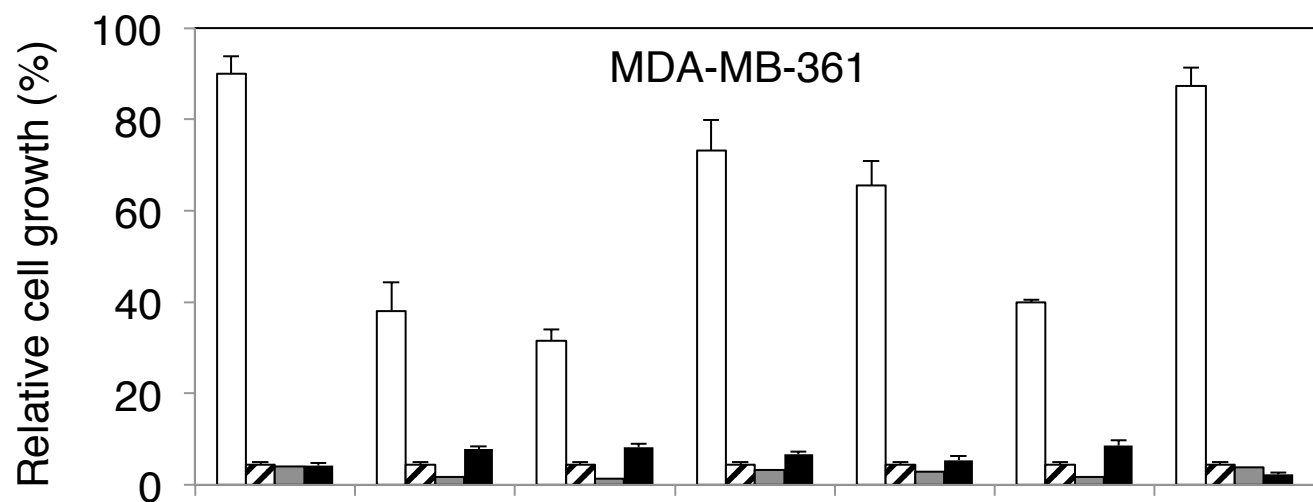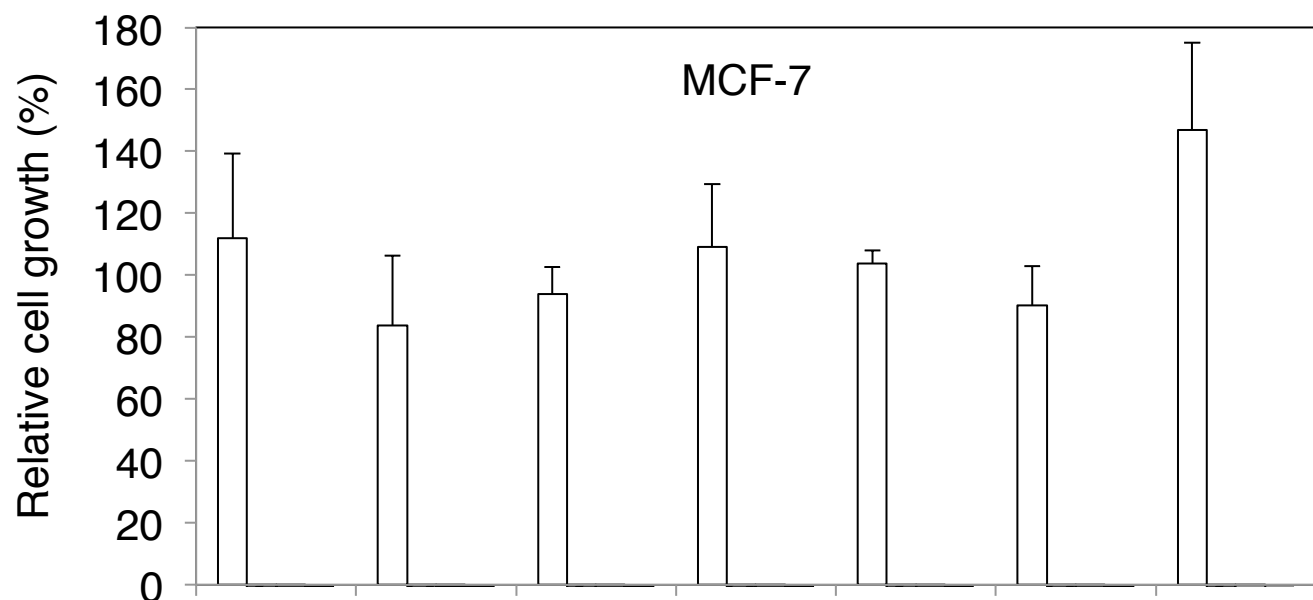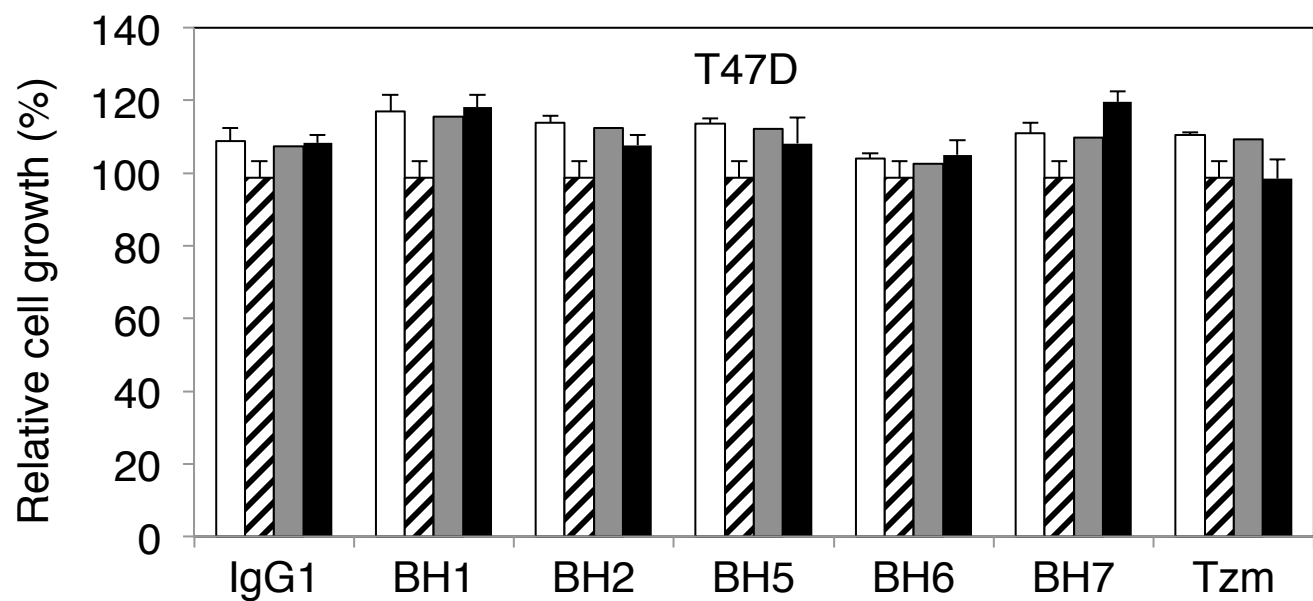

Supplement: Additional file 4 — Interaction between anti-HER2 antibodies and tumor necrosis factor-α. MDA-MB-361 cells were partially sensitive to anti-HER2 antibodies, but highly sensitive to TNF-α (top). MCF-7 cells were resistant to anti-HER2 antibodies, but highly sensitive to TNF-α (middle). T47D cells were resistant to both anti-HER2 antibodies and TNF-α (bottom). Growth measurements observed under 5μg/ml antibody (white columns), 1000 U/ml TNF-α (striped columns), and 5 μg/ml antibody + 1000 U/ml TNF-α (black columns) were obtained experimentally, as described in Figure 6. Growth level under a drug condition was defined as the growth under that condition normalized by growth under no drug condition. Expected growth level under no interaction (gray columns) was calculated by multiplying the growth levels under each individual drug. The observed growth level for each combination was divided by the expected growth level to find an interaction score according to Bliss Independence Model for drug interactions. Interaction scores were not calculated for these cell lines. [file 1471-2407-12-450-S4.pdf]

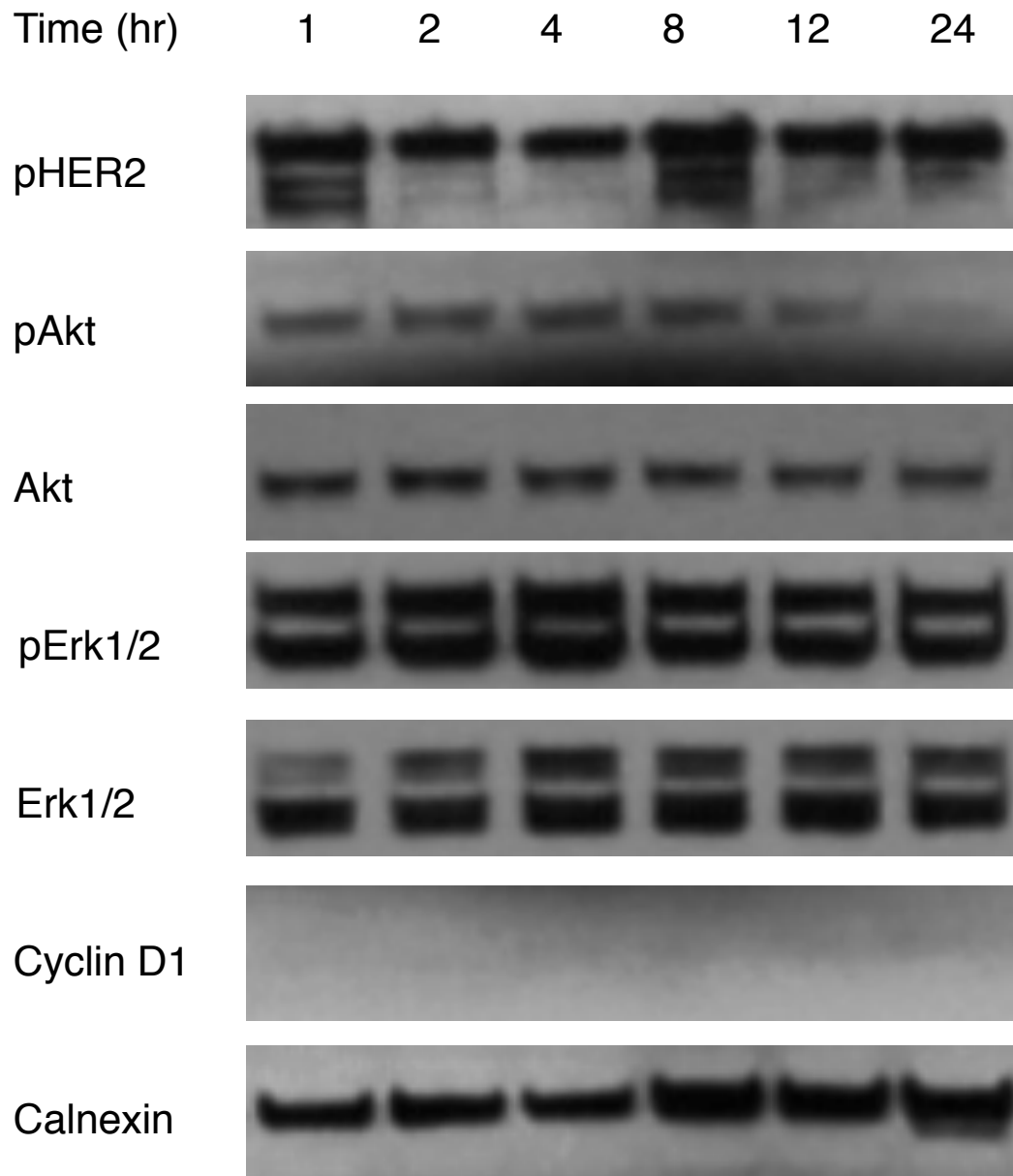

Supplement: Additional file 5 — In vitro molecular responses of SK-BR-3 cells to tumor necrosis factor-α. SK-BR-3 cells were treated up to 24 h with TNF-α (1000 U/ml). Cell lysates were prepared from cells harvested at indicated times (hr, hours) and the expressions of phospho-HER2 (pHER2), phospho-Akt (pAkt), total Akt (Akt), phospho-ERK1/phospho-Erk2 (pErk1/2), total Erk1/Erk2 (Erk 1/2) and Cyclin D1 were analyzed by western blotting. Calnexin was used as a loading control. [file 1471-2407-12-450-S5.pdf]
